# Evolution of appraisal tool usage preferences in PROSPERO records: a study of non-Cochrane systematic reviews

**DOI:** 10.1186/s12874-023-02114-0

**Published:** 2023-12-14

**Authors:** J. Ruano, J. Gay-Mimbrera, M. Aguilar-Luque, F. Gómez-García, E. Parra-Peralbo, B. Isla-Tejera

**Affiliations:** 1 Immune-mediated Inflammatory Skin Diseases group, IMIBIC/Reina Sofía University Hospital/University of Cordoba, Córdoba, 14004 Spain; 2 Department of Dermatology, Reina Sofía University Hospital, Córdoba, 14004 Spain; 3 Faculty of Biomedical Science and Health, Universidad Europea, Madrid, 28108 Spain; 4 Department of Pharmacy, Reina Sofía University Hospital, Córdoba, 14004 Spain

**Keywords:** Research-on-research, Methodological appraisal tools, Protocols, Systematic reviews, Methodological quality, PROSPERO repository

## Abstract

**Objectives:**

This research-on-research substudy uses a data-driven approach to investigate the range of appraisal tools in non-Cochrane systematic reviews and meta-analyses registered in the International Prospective Register of Systematic Reviews (PROSPERO).

**Study design and setting:**

A comprehensive web scraping of all completed non-Cochrane registrations in PROSPERO from February 2011 to December 2017 was performed. The focus was classifying the appraisal tools based on study type, assessment aspects, and research topics.

**Results:**

After analyzing 17,708 complete records, we found a predominant use of methodological quality assessment tools compared to those for reporting quality or risk of bias (RoB). This indicates a greater emphasis on methodological rigor in the studied protocols. Various tools for assessing methodological quality were observed, reflecting the complexity of such evaluations. Instruments designed for evaluating methodological or reporting quality were mainly intended for non-randomized clinical trials or observational studies, unlike RoB tools more commonly used in randomized clinical trials. No distinct trends in tool usage were observed in specific research conditions or domains, suggesting that tool choice is influenced more by study design than research topic.

**Conclusion:**

This study provides insights into the preferential use of various assessment tools in conducting non-Cochrane systematic reviews, as evidenced in PROSPERO records. The findings reveal various methodological assessment tools, underscoring their versatility across different study designs and research areas.

## Introduction

The International Prospective Register of Systematic Reviews (PROSPERO) was established and is maintained by the UK Center for Reviews and Dissemination (CRD) at the University of York in England (https://www.crd.york.ac.uk/PROSPERO/). The purpose of creating a protocol with an *a priori* definition of the research question, eligibility criteria, and data analysis methods is to promote transparency in the review process, minimize reporting bias, and prevent unnecessary duplication of reviews. Researchers are encouraged to register their reviews in PROSPERO or publish the protocol in a scientific journal, providing updates and modifications based on reviewer suggestions.

PROSPERO has become the preferred platform for registering systematic review (SR) protocols, offering a template with multiple fields covering various aspects of the protocol [1]. The field designated for *risk of bias (RoB) assessment* allows reviewers to outline their chosen approaches and appraisal tools for evaluating the quality, RoB, and reporting quality of primary studies [2].

The concept of methodological quality in SRs refers to the likelihood of achieving the highest possible standard in the review process and may overlap with the notion of bias risk. The Cochrane Handbook for SRs of Interventions distinguishes between assessments of methodological quality and RoB assessments [3]. Our research has demonstrated that it is feasible to develop a review with high methodological quality yet still have a high RoB [4].

Various scales, checklists, and domain-based tools are available to appraise SRs critically. Previous studies by Moher et al. in 2004 [5] and Page et al. in 2014 [6] explored the epidemiology and reporting characteristics of SRs, comparing Cochrane and non-Cochrane reviews using random samples of 300 published reviews. They found that 70% of the reviews utilized tools to assess the quality (methodology and reporting) and RoB of primary and secondary studies. The most commonly used tool was the Cochrane RoB tool, employed in 80% of Cochrane reviews and 41% of non-Cochrane reviews. The Jadad and Newcastle-Ottawa (NOS) scales were also frequently used. For SRs focusing on diagnostic methods, only 40% of studies utilized a validated tool such as the Quality Assessment of Diagnostic Accuracy Studies (QUADAS/QUADAS-2) tool, with QUADAS being the most prevalent at 32%. However, the reasons for selecting these different tools listed in the PROSPERO records have not been described.

This study aims to analyze the evolution of tool usage frequency and assess the appropriateness of tool selection to study design and the specific condition or domain being analyzed. To achieve this, we examined the entire collection of non-Cochrane SR records in the PROSPERO repository since its establishment.

## Methods

This research, conceived as a substudy within a research-on-research protocol, aligns with our broader project’s scope as outlined in the 2018 *a priori* protocol [7]. It specifically examines the application of methodological tools in systematic review protocols registered in PROSPERO. Strategically selecting a study period up to the year 2017, our approach enables a detailed 5-year post-protocol analysis, aiming to track the publication outcomes of these reviews. This defined timeframe is practical and critical for evaluating evolving methodological trends and tool utilization in systematic reviews.

Our protocol lays the groundwork for this study, outlining the data source, the methodology for document retrieval (including PROSPERO web scraping), the criteria for eligibility and screening, and procedures for data extraction, analysis, and reporting. This protocol provides a comprehensive framework, ensuring a robust and transparent methodological approach for our current analysis.

### Web scraping strategy

The records stored in PROSPERO (http://www.crd.york.ac.uk/prospero/) were obtained through web scraping techniques. A custom Python 3.0 script and the Chrome web scraper website data extraction tool (http://webscraper.io/) were used to automate raw data extraction from all completed non-Cochrane registration records. The data was collected from February 2011 to December 2017 iteratively.

### Data filtering and eligibility criteria

Registers or protocols that had less than 90% of the fields completed or duplicated (i.e., sharing titles and reviewers) were excluded from the dataset. This screening process was automated using an R script. The remaining results underwent a human verification process conducted by two reviewers, JG-M and MA-L, to ensure accuracy and consistency.

### Dataset and variables

The above steps generated a working *.csv file containing only the relevant variables. In cases where protocols involved multiple reviewers from different countries, they were considered the outcome of an international collaboration, and the respective countries were grouped, contributing to collaborative protocols. Using text-mining techniques followed by human supervision, a unique condition or domain of study was assigned to each protocol. The appraisal tools were categorized based on their intended purpose, such as assessing RoB, reporting quality, or methodological quality. When a single institution provided a selection of tools that could be used for multiple assessment tasks, these tools were classified as an appraisal toolkit. The variable indicating the study design was determined based on the nature of the primary studies specified in the inclusion criteria. The occurrence patterns of the tools in the protocols were analyzed, considering the purpose of the appraisal, the study design, and the year of registration in PROSPERO.

### Data visualization and statistical analysis

Graphs were produced, and statistics were analyzed using several packages available for the R 3.4.4 language [R Development Core Team (http://www.R-project.org)], except for the workflow diagram, which was created using Review Manager 5 (RevMan 5) software (https://community.cochrane.org/help/tools-and-software/revman-5).

### Changes from the protocol

As previously mentioned, our search strategy was published and compared with the final reported review methods [7]. The methods of web scraping, filtering, and selection remained unchanged from the previous report to this one. However, it is essential to note that this article only briefly describes the procedure as it is the second article in the series. We encourage readers to refer to the abovementioned protocol for a more comprehensive understanding of the methodology.

## Results

In collecting and filtering the PROSPERO records, 30,000 were initially scraped. However, 5,362 records were excluded due to incompleteness, and 903 were identified as duplicates of other protocols. After text mining, 4,822 documents were excluded, primarily due to blank records or lacking essential information.

Human-supervised filtering excluded 4,364 records due to duplications or missing data. Furthermore, 2,182 records were excluded because they lacked information in the “RoB (quality) assessment” field.

**Fig. 1:**
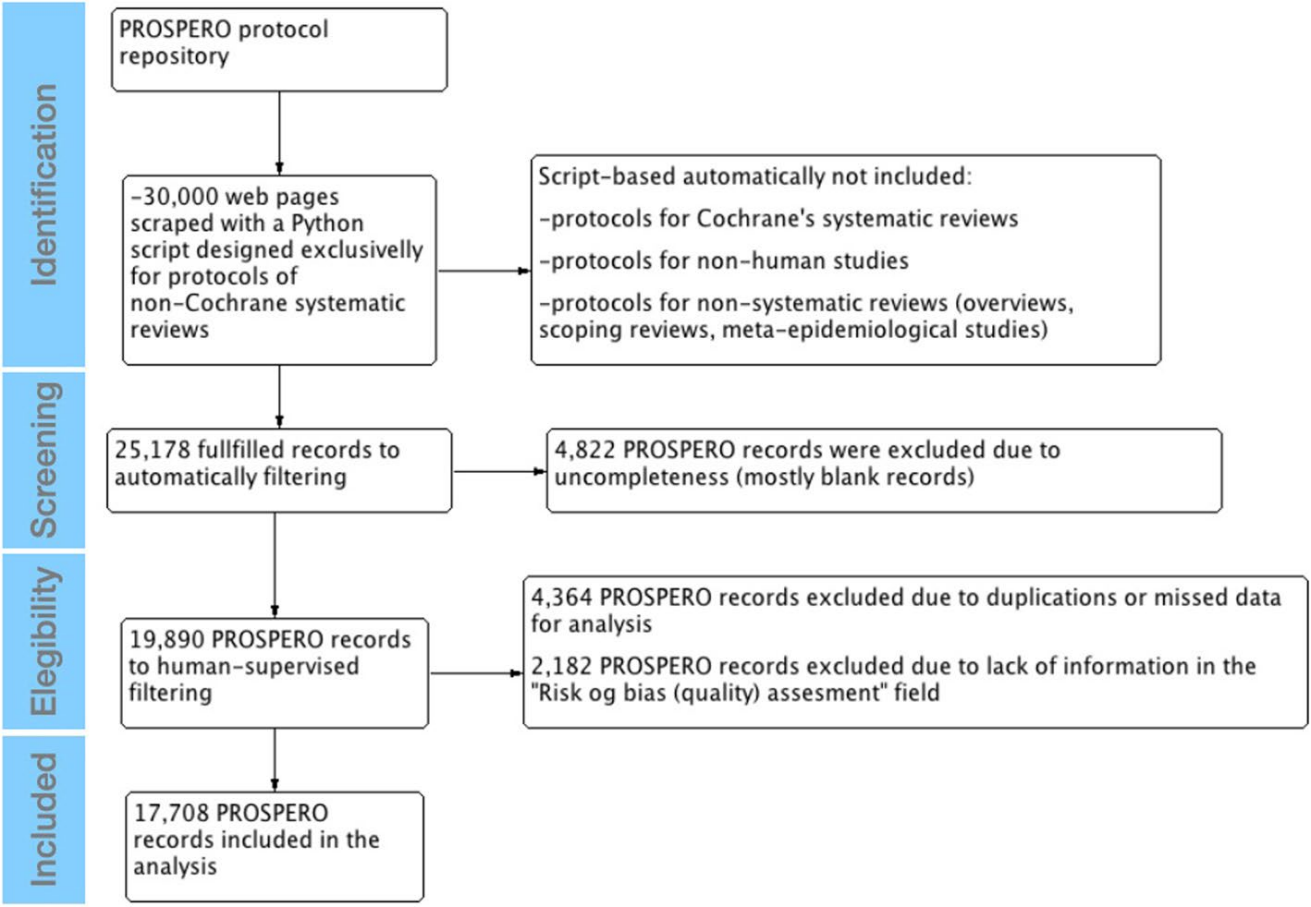
PRISMA workflow of searching for PROSPERO records and protocols published in scientific journals about non-Cochrane systematic reviews

After removing the excluded records, 17,708 PROSPERO records from 2011 to 2017 remained available in subsequent analyses. The overall process and exclusions are illustrated in Fig. 1, providing an overview of the data collection and filtering stages.

### General characteristics

Our review of PROSPERO records identified 49 distinct appraisal tools and toolkits, as detailed in Tables 1 and 2. Collectively, these tools were referenced 10,215 times across the records. The primary application of these tools was for assessing methodological quality, with 5,170 mentions in the protocols accounting for 50.6% of the total. This was followed by tools for evaluating the risk of bias (29.4%) and those assessing the quality of reporting (9.4%).

**Table 1:** Frequency of methodological quality appraisal tools in non-Cochrane systematic reviews registered in PROSPERO (2011-2017)

Tool	n (%)	Descriptive
**Methodological quality**	5,170 (50.6%)	
*Newcastle-Ottawa scale (NOS)*	1,823 (5.5%)	Newcastle-Ottawa quality assessment scale for case-control studies.
*GRADE*	983 (9.6%)	The Grading of Recommendations Assessment, Development and Evaluation (GRADE) approach.
*Jadad*	467 (5.5%)	Jadad quality assessment scale for rating randomized controlled trials.
*PEdro scale*	432 (5.5%)	A scale to measure the quality of reports of randomized controlled trials indexed on the Physiotherapy Evidence Database(PEDro).
*Downs and Black check-list*	323 (5.5%)	Tool for assessing the methodological quality of both randomized and non-randomized studies.
*AMSTAR*	314 (5.5%)	A Measurement Tool to Assess Systematic Reviews (AMSTAR) methodological quality.
*EPHPP*	158 (5.5%)	The Effective Public Health Practice Project (EPHPP) assessment tool for quantitative studies.
*COSMIN checklist*	139 (5.5%)	COnsensus-based Standards for the selection of health status Measurement INstruments (COSMIN)checklist evaluates studies on measurement properties.
*MINORS*	128 (2.4%)	Methodological Index for Non-Randomized Studies (MINORS) was designed to assess the non-randomized surgical studies, whether comparative or non-comparative.
*MMAT*	85 (1.6%)	Mixed Methods Appraisal Tool (MMAT) for qualitative, quantitative, and mixed method studies.
*McMaster*	76 (1.4%)	Quality assessment tool for quantitative studies.
*Drummond checklist*	54 (1.04%)	Tool for assessing economic evaluations.
*AGREEII*	45 (0.87%)	The Appraisal of Guidelines for Research and Evaluation (AGREE) II instrument for clinical practice guidelines.
*CHEERS*	42 (0.81%)	ISPOR’s Consolidated Health Economic Evaluation Reporting Standards (CHEERS).
*CMS/MCMS*	24 (0.46%)	
*MERSQI*	19 (0.36%)	The Medical Education Research Study Quality Instrument (MERSQI)to appraise methodological quality in medical education research.
*QATSDD*	19 (0.36%)	Quality Assessment Tool for reviewing Studies with Diverse Design (QATSDD) for quantitative and qualitative studies.
*Phillips checklist*	15 (0.29%)	A tool for the appraisal of the methodological quality of economic modeling studies
*QATSO*	12 (0.23%)	Quality Assessment Tool for Systematic Reviews of Observational Studies (QATSO) score.
*Q-Coh*	11 (0.21%)	A tool to screen the methodological Quality of CoHort studies in systematic reviews and meta-analyses.
*ISPOR*	7 (0.13%)	International Society for Pharmacoeconomics and Outcomes Research (ISPOR) checklist.
*OQAQ*	7 (0.13%)	Overview Quality Assessment Questionnaire (OQAQ) was designed to assess the methodological quality of SRs.
*CHEC list*	6 (0.11%)	The Consensus Health Economic Criteria (CHEC) for assessment of the methodological quality of economic evaluations.
*RE-AIM*	6 (0.11%)	The RE-AIM framework was designed to enhance the quality, speed, and public health impact of efforts to translate research into practice.
*SAQOR*	5 (0.09%)	Systematic Assessment of Quality in Observational Research (SAQOR) for systematic reviews of observational studies.
*SEQES*	3 (0.03%)	Structured Effectiveness Quality Evaluation Scale (SEQES).
*AQUA*	2 (0.02%)	The Anatomical Quality Assurance (AQUA) checklist was developed to improve the quality of reporting of anatomical studies.
*GRACE*	2 (0.02%)	Good ReseArch for Comparative Effectiveness (GRACE) checklist is designed for the quality assessment of observational studies of comparative effectiveness.

**Table 2:** Distribution of risk of bias, reporting quality, and critical appraisal tools in non-Cochrane systematic reviews in PROSPERO (2011-2017)

Tool	n (%)	Descriptive
**Risk of bias**	3,012 (29.4%)	
* Cochrane Risk of Bias*	2,092 (20.5%)	
* QUADAS/QUADAS-2*	552 (5.4%)	Quality Assessment of Diagnostic Accuracy Studies (QUADAS) is a tool for the quality assessment of diagnostic accuracy studies.
* ROBINS-I*	189 (1.8%)	Risk Of Bias In Non-randomized Studies - of Interventions (ROBINS-I).
* QUIPS*	100 (0.9%)	Quality In Prognosis Studies (QUIPS) tool.
* ROBIS*	43 (0.4%)	Tool to assess Risk Of Bias In Systematic reviews (ROBIS).
* RoBANS*	33 (0.3%)	Risk of Bias Assessment tool for Non-randomized Studies (RoBANS).
* ReBIP*	3 (0.03%)	Review Body for Interventional Procedures (ReBIP).
**Reporting quality**	969 (9.4%)	
* STROBE*	410 (4.1%)	Strengthening the reporting of observational studies (cohort, case-control, and cross-sectional).
* PRISMA*	285 (2,8%)	Preferred Reporting Items for Systematic Reviews and Meta-Analyses (PRISMA)
* CONSORT*	156 (1.5%)	Consolidated Standards of Reporting Trials (CONSORT).
* COREQ*	48 (0.4%)	Consolidated criteria for reporting qualitative research (COREQ).
* MOOSE*	39 (0.4%)	Meta-analyses of Observational Studies (MOOSE) check-list.
* STARD*	24 (0.2%)	Standards for the Reporting of Diagnostic (STARD) accuracy studies.
* CLEAR NPT*	4 (0.04%)	Check list for reporting nonpharmacological trials.
* SRQR*	2 (0.02%)	Standards for Reporting Qualitative Research (SRQR).
* QUOROM*	1 (0.1%)	Quality of Reporting of Meta-analyses (QUOROM).
**Critical Appraisal Toolkits**	1,064 (10.4%)	
* CASP Critical Appraisal Tools*	571 (5.5%)	Critical Appraisal Skills Programme (CASP) tool.
* JBI Critical Appraisal Tools*	373 (5.5%)	Joanna Briggs Institute (JBI) Critical Appraisal tools.
* SIGN Critical Appraisal Tools*	46 (0.45%)	Scottish Intercollegiate Guidelines Network (SIGN) Critical Appraisal notes and checklists.
* CEBM Critical Appraisal Tools*	39 (0.4%)	Centre for Evidence-Based Medicine (CEBM) critical appraisal tools.
* BMJ Critical Appraisal Tools*	35 (0.34%)	British Medical Journal (BMJ) critical appraisal tools.

Within the 9,676 records that provided detailed information about the appraisal tools and study types, the most commonly planned studies were RCTs, noted in 3,316 records (34.2%). Non-RCTs and observational studies were also frequent (29%). Additionally, a combination of RCTs and observational studies was planned in 326 records (3.3%). Other primary study types included diagnostic accuracy studies (6.3%), economic evaluation studies (1.2%), qualitative studies (0.5%), quantitative studies (1.63%), mixed-design research studies (0.16%), patient-reported outcome measures studies (1.4%), and medical education studies (0.2%).

The analysis also revealed that some records incorporated tools for evaluating secondary studies. These included meta-epidemiological or research-on-research studies about clinical practice guidelines (15%), systematic reviews, and meta-analyses (6.7%).

### Frequency of tools by appraisal objective

In our analysis of PROSPERO records, 27 tools were identified for assessing methodological quality, as detailed in Table 1. The Newcastle-Ottawa Scale (NOS) (35.2%) was prominent among these, primarily utilized for evaluating case-control studies. Other notable instruments included the Grading of Recommendations, Assessment, Development, and Evaluation (GRADE), the Jadad scale, and the Physiotherapy Evidence Database (PEDro) scale.

Seven distinct tools were recognized to assess the RoB (Table 2). The Cochrane tool for RCTs was most commonly referenced (20.5%). This was followed by the Quality Assessment of Diagnostic Accuracy Studies (QUADAS/QUADAS-2) tool, the RoB in Non-randomized Studies of Interventions (ROBINS-I) instrument, and the Quality In Prognosis Studies (QUIPS) tool.

Nine tools were identified in reporting quality (Table 2). The Strengthening the Reporting of Observational Studies in Epidemiology (STROBE) tool, aimed at reporting observational studies, was frequently mentioned (20.5%). Others included the Preferred Reporting Items for Systematic Reviews and Meta-Analyses (PRISMA) checklist, the Consolidated Standards of Reporting Trials (CONSORT) for randomized trials, and the Meta-Analyses of Observational Studies (MOOSE) guideline.

Additionally, 10.4% of the records mentioned use critical appraisal toolkits developed by various global institutions catering to multiple appraisal needs (Table 2). The Critical Appraisal Skills Programme (CASP) and the Joanna Briggs Institute (JBI) toolkits were among the most referenced in this category.

### Frequency of tools by appraisal objective and condition or domain being studied

**Fig. 2:**
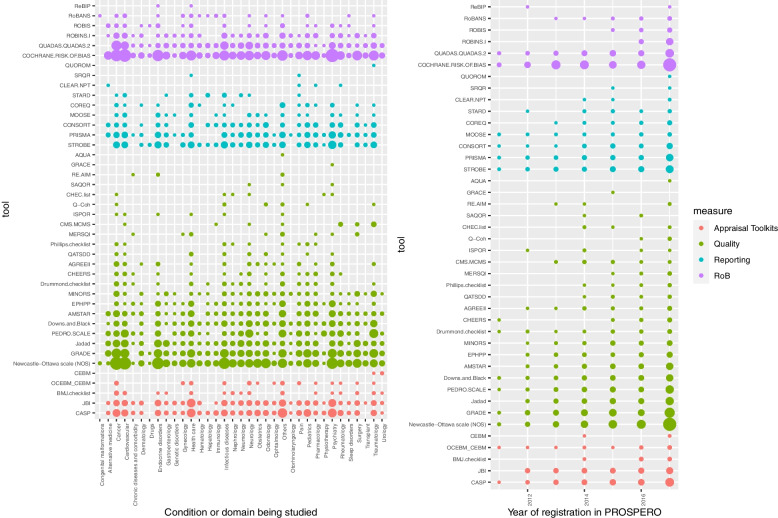
This panel represents tools included in protocols for SRs by condition or domain being studied and time-course changes. **a** Tools by condition or domain being studied. Points represent protocols and are colored according to the appraisal purpose of the toolkit: red for risk of bias tools, blue for reporting assessment tools, green for methodological quality tools, and black for multiple-purpose appraisal tools. Point size is proportional to the number of protocols in each category. **b** Tools by year of registration in PROSPERO

The appraisal tools in our study were categorized based on their objectives, as illustrated in Fig. 2a. The frequency of use for each tool was analyzed according to the condition or domain targeted by the respective protocol. The most frequently studied disorders were psychiatry, cancer, and cardiovascular disease. Conversely, areas like chronic diseases and comorbidity, drugs, and congenital malformations were among the least studied.

Figure 2b depicts the temporal patterns of tool usage, tracking their inclusion in PROSPERO records by year of registration. This graph indicates a progressive increase in the incorporation of appraisal tools in protocols, with a notable peak observed in 2016 and 2017. However, the usage patterns varied across different periods.

The analysis revealed a consistent rise in the inclusion of tools for methodological quality assessment in PROSPERO records from 2011 to 2017. This increase surpassed the frequency of tools used for RoB and reporting quality assessment. The diversity of tools for various appraisal purposes also expanded, reflecting the development of new tools over this period. In 2011, a limited number of tools were mentioned, while by 2017, this number had significantly increased.

Earlier, specific tools like the Cochrane tool and the QUADAS checklist were primarily used for assessing RoB. In contrast, tools like STROBE, the PRISMA checklist, MOOSE, and CONSORT were noted for reporting quality appraisal. Tools such as the NOS, GRADE, Downs and Black checklist, and the PEDro scale were mentioned for methodological quality appraisal, along with several others. Toolkits like the Critical Appraisal Skills Programme (CASP) and the Center for Evidence-Based Medicine (CEBM) were also included in the records.

By 2017, the number of appraisal tools in PROSPERO records had increased across all categories (Fig. 2b), with tools such as the NOS, the Cochrane tool for RoB, GRADE, and the CASP toolkit being among the most frequently mentioned.

These findings underscore the growing use of appraisal tools in systematic review protocols, especially for assessing methodological quality. The increasing variety of tools reflects the evolution and introduction of new instruments, contributing to a more thorough and nuanced evaluation of research quality.

### Tools and study design

**Fig. 3:**
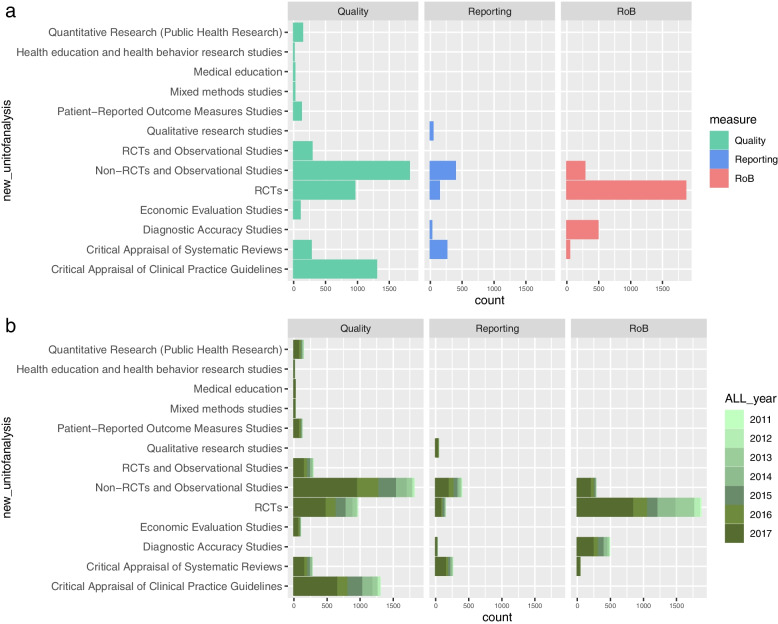
**a** Protocols by study design and tool’s appraisal purpose. **b** Protocols by tool and study design considering the year of registration in PROSPERO

**Fig. 4:**
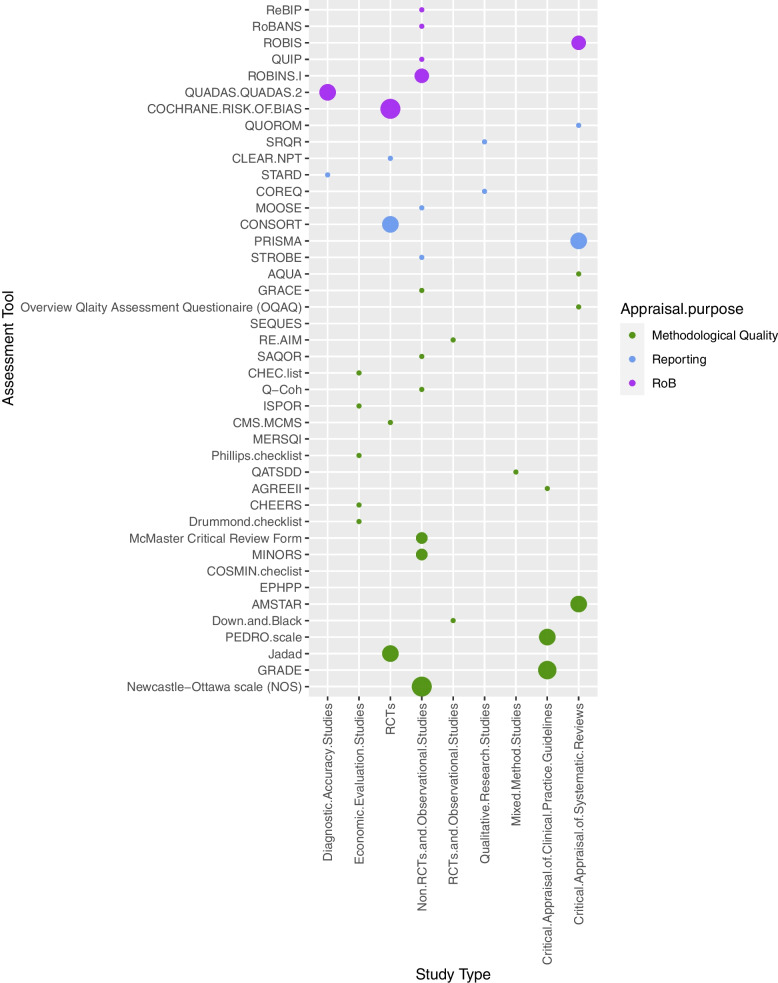
Overall frequency of appraisal tools included in PROSPERO records by primary study design according to the tool’s appraisal purpose

In our study, we categorized the protocols based on the primary study design and the purpose of the appraisal tools, as shown in Figs. 3a, b, and 4. The analysis indicated that tools for assessing methodological and reporting quality were primarily used in SR protocols focusing on non-RCTs and observational studies. In contrast, RoB analysis tools were predominantly found in protocols for randomized controlled trials (RCTs).

Protocols involving critical appraisal of SRs displayed a more excellent representation of tools for assessing methodological and reporting quality than those for RoB evaluation. Notably, the integration of RoB evaluation tools in protocols became more apparent after 2017, a trend illustrated in Fig. 3b.

In the context of economic evaluations and clinical practice guideline protocols, the primary focus was assessing methodological quality. Meanwhile, in diagnostic accuracy study protocols, the emphasis was on RoB evaluation, with less on reporting and methodological quality.

These findings highlight the varying emphasis on different appraisal tools across diverse study designs and research domains. While methodological and reporting quality assessment tools were more prevalent in SRs of non-RCTs and observational studies, RoB evaluation tools gained prominence in RCTs. The increasing inclusion of RoB evaluation tools in recent years underscores an evolving recognition of the importance of bias assessment in research synthesis.”

## Discussion

### Main findings

#### Our findings in context

Our recent study delved into the patterns of using scientific quality assessment tools in systematic review (SR) protocols, as reported in PROSPERO between 2011 and 2017. We observed a notable preference for methodological quality assessment tools in these protocols over those used for evaluating reporting quality or RoB. This trend suggests an overarching emphasis on methodological robustness in the examined protocols.

Further analysis revealed a diverse range of appraisal tools for assessing methodological quality, more so than for reporting quality or RoB. This diversity underscores a broader acknowledgment of the complexities inherent in methodological evaluation. Interestingly, we found that most tools used for evaluating methodological or reporting quality were primarily designed for non-randomized clinical trials or observational studies. In contrast, tools for RoB assessment were more often developed with randomized clinical trials in mind, reflecting the distinct needs and challenges of different study designs.

Moreover, our study did not identify any distinct patterns in using these tools based on the specific condition or domain of research. This finding suggests that the selection of appraisal tools is likely more influenced by the study design rather than the research topic or condition under investigation.

These insights contribute significantly to understanding current preferences and trends in using appraisal tools in SR protocols. They also shed light on the focus areas and the range of tools employed for quality assessment in these studies. By highlighting how methodological tools are chosen and applied in systematic reviews, our analysis underscores the dynamic nature of research evaluation practices in the evolving landscape of scientific inquiry.

#### Limitations and strengths

Our study thoroughly analyzed non-Cochrane systematic review (SR) protocols registered in PROSPERO from 2011 to 2017. We specifically selected 2017 as the endpoint for data collection to align with our primary goal of determining which protocols led to published studies. This timeframe was chosen for its practicality in managing a comprehensive dataset and enabling a 5-year follow-up analysis post the latest 2017 protocol, providing insights into the transition from protocol to publication.

This approach is crucial for understanding systematic review trends over time despite limiting the inclusion of more recent studies. Our analysis, grounded in a previously established *a priori* protocol [7], examines the entire set of PROSPERO registrations from this period, ensuring a complete and unbiased view of the trends in systematic review protocols. We employed a Python script designed to analyze non-Cochrane PROSPERO records, achieving a sensitivity and specificity of 100% in our web scraping approach.

Our research aligns with previous findings that no single tool covers all elements of internal validity, emphasizing the need for careful selection based on study design [8–11]. For example, Santiago-Delefosse et al. identified 133 qualitative appraisal tools developed between 1985 and 2014 [12].

However, our study has limitations. There’s a possibility that some tools used for evidence appraisal might have been missed, although extensive screening reduces this likelihood. Nevertheless, the possibility of missing important and frequently employed tools is low, considering we extensively screened various data sources, including the Internet. Furthermore, specific protocols mentioned using unspecified or homegrown tools were excluded due to transparency issues regarding their validity.

While acknowledging these limitations, it’s important to note that our method only analyzes the planned methodologies in the protocols, which might differ from the actual conduct of the reviews. The ideal approach would involve comparing the designed tools in the protocols with those used in the published reviews. However, a comprehensive analysis of published reviews is computationally challenging due to the diversity in article structures and the volume of documents involved. Future research could explore these aspects further, potentially offering a more holistic view of the methodologies employed in systematic reviews.

#### Implications of results

Critical appraisal is essential in evaluating clinical research papers for their internal and external validity. The development of appraisal tools plays a crucial role in this process, validating aspects such as methodological quality, risk of bias, and adherence to reporting standards. These tools are pivotal in providing clinicians, researchers, patients, and policymakers with confidence in research findings, enabling informed decision-making based on the quality and reliability of these results.

The evolution of appraisal tools reflects the progression of empirical evidence. As research methodologies advance, outdated tools are often replaced or updated to align with contemporary standards. For example, the PRISMA checklist superseded the QUOROM scale in 2009 for evaluating reporting quality [13], and the OQAQ evolved into AMSTAR [14], ROBIS [15], and subsequently AMSTAR2 over the years2017 [16]. These changes illustrate the dynamic nature of appraisal tools in adapting to new research designs and methodologies.

Our retrospective analysis included outdated and current versions of these tools as they appeared in their protocols. This approach allowed us to track the usage of various instruments over time and observe shifts in their adoption. However, it limited our ability to explore specific analyses, such as the resistance to adopting new appraisal tools once they are introduced.

Critical appraisal tools are indispensable in guiding researchers through objectively evaluating reported results in evidence synthesis studies. Their goal is to ensure consistency in conclusions and focus on study design elements that significantly impact the credibility of results. These tools, developed as part of standards for systematic reviews, aim to enhance transparency, reproducibility, and methodological rigor over time. Rooted in principles of epidemiological study design and supported by empirical evidence or expert consensus, these standards underscore the unchanging core principles of critical appraisal despite the evolution in tool usage.

## Conclusions

Our study provides a detailed investigation into the preferred use of assessment tools in non-Cochrane systematic reviews based on PROSPERO records. The findings reveal various methodological assessment tools, highlighting their applicability across different studies. This research contributes to a deeper understanding of the diverse tools available for conducting systematic reviews and their specific uses in research methodology.

## Data Availability

The datasets generated and analyzed during the current study are available in the GitHub repository, including raw data and Python and R scripts in our [GitHub Repository].
